# The threat of the COVID-19 pandemic on reversing global life-saving gains in the survival of childhood cancer: a call for collaborative action from SIOP, IPSO, PROS, WCC, CCI, St Jude Global, UICC and WHPCA

**DOI:** 10.3332/ecancer.2021.1187

**Published:** 2021-02-15

**Authors:** Kathy Pritchard-Jones, Simone de C V Abib, Natia Esiashvili, Gertjan J L Kaspers, Jon Rosser, John A Van Doorninck, João M L Braganca, Ruth I Hoffman, Carlos Rodriguez-Galindo, Cary Adams, Stephen R Connor, Abdelhafeez H Abdelhafeez, Eric Bouffet, Scott C Howard, Julia M Challinor, Laila Hessissen, Rashmi B Dalvi, Pamela Kearns, Guillermo L Chantada, Lindsay A Frazier, Michael J Sullivan, Fiona S M Schulte, Lisa K Morrissey, Olga Kozhaeva, Sandra Luna-Fineman, Muhammad S Khan

**Affiliations:** 1 UCL Great Ormond Street Institute of Child Health, University College London, 30 Guilford Street, London WC1N 1E, United Kingdom; 2 International Society of Paediatric Oncology (SIOP), Industriestrasse 25, 6312 Steinhausen, Switzerland; 3 Division of Pediatric Surgery, Pediatric Oncology Institute (GRAACC), Federal University of São Paulo, Rua Pedro de Toledo, 572, 04039-001 São Paulo, Brazil; 4 International Society of Paediatric Surgical Oncology (IPSO), Tienrayseweg 10, 5961NL Horst, The Netherlands; 5 Department of Radiation Oncology, Winship Cancer Institute at Emory University, 1365 Clifton Road, NE, Atlanta, GA 30322, USA; 6 Paediatric Radiation Oncology Society (PROS), 28 rue Laennec, F-69373 Lyon, Cedex 08, France; 7 Academy and Outreach, Princess Máxima Center for Pediatric Oncology, Heidelberglaan 25, NL-3584 CS Utrecht, The Netherlands; 8 Department of Pediatric Oncology, Emma Children’s Hospital, Amsterdam UMC, Vrije Universiteit Amsterdam, De Boelelaan 1117, 1118, 1081 HV Amsterdam, The Netherlands; 9 World Child Cancer (WCC), The Netherlands, P.O. Box 113, 3720 AC Bilthoven, The Netherlands; 10 World Child Cancer (WCC) UK, 9 Maltings Place, London SE1 3JB, UK; 11 Department of Pediatrics, Division of Pediatric Hematology/Oncology, Rocky Mountain Hospital for Children, 2055 High Street, #340, Denver, CO 80211, USA; 12 World Child Cancer (WCC) USA, 1301 Arapahoe St Suite 105, Golden, CO 80401, USA; 13 Childhood Cancer International (CCI), Kraijenhoffstraat 137A, 1018RG Amsterdam, The Netherlands; 14 Department of Global Medicine, St Jude Children’s Research Hospital, 262 Danny Thomas Place, Memphis, TN 38105, USA; 15 Union for International Cancer Control (UICC), 31-33 Avenue Giuseppe Motta, 1202 Geneva, Switzerland; 16 Worldwide Hospice Palliative Care Alliance (WHPCA), 10990 Rice Field Pl, Fairfax Station, VA 22039, USA; 17 Department of Surgery, St. Jude Children’s Research Hospital, 262 Danny Thomas Place, Memphis, TN 38105, USA; 18 Division of Haematology/Oncology, University of Toronto, The Hospital for Sick Children, 555 University Ave, M5G 1X8, Toronto, Canada; 19 Department of Acute and Tertiary Care, University of Tennessee Health Science Center, Memphis, TN 38103, USA; 20 School of Nursing, University of California San Francisco, 36 Rockview Dr., Santa Cruz, CA 95062, USA; 21 Department of Pediatric Hematology and Oncology, Children Hospital of Rabat, Mohamed V university of Rabat, Ibn Rochd avenue, Rabat 6542, Morocco; 22 Department of Pediatrics and Pediatric Hematology Oncology, Bombay Hospital Institute of Medical sciences, street 20, New Marine lines, Mumbai 400020, India; 23 Institute of Cancer and Genomic Sciences, NIHR Birmingham Biomedical Research Centre, University of Birmingham, Vincent Drive, Birmingham B15 2TT, UK; 24 European Society for Paediatric Oncology (SIOP Europe), Clos Chapelle-aux-Champs 30, 1200 Brussels, Belgium; 25 Department of Hematology-Oncology, Hospital Pereira Rossell-Fundacion Perez-Scremini (secondary: Hospital Sant Joan De Deu), Bulevar Artigas 1556, 11600 Montevideo, Uruguay; 26 Department of Pediatric Oncology, Dana-Farber Cancer Institute, 450 Brookline Ave, Boston, MA 02115, USA; 27 Children’s Cancer Centre and Department of Paediatrics, Royal Children’s Hospital and University of Melbourne, 50 Flemington Road, Parkville 3052, Melbourne, Australia; 28 Paediatric Oncology in Developing Countries (PODC) Committee, International Society of Paediatric Oncology (SIOP), Industriestrasse 25, 6312 Steinhausen, Switzerland; 29 Department of Oncology, Division of Psychosocial Oncology, University of Calgary, 2202 2 St., T2S 3C3, Calgary, Canada; 30 Paedatric Psycho-Oncology (PPO) Committee, International Society of Paediatric Oncology (SIOP), Industriestrasse 25, 6312 Steinhausen, Switzerland; 31 Nursing and Patient Services, Division of Hematology/Oncology/HSCT, Boston Children’s Hospital, 300 Longwood Avenue, Boston, MA 02115, USA; 32 Nursing Committee, International Society of Paediatric Oncology (SIOP), Industriestrasse 25, 6312 Steinhausen, Switzerland; 33 Policy Affairs, European Society for Paediatric Oncology (SIOP Europe), Clos Chapelle-aux-Champs 30, 1200 Brussels, Belgium; 34 Division of Hematology/Oncology/SCT, Department of Pediatrics, Children’s Hospital Colorado, U Colorado, 13123 E 16th Ave, B115, Aurora, CO 80045, USA; 35 Pediatric Hematology and Oncology Division, Tawam Hospital, Al Ain (Abu Dhabi), PO Box 15258, United Arab Emirates

**Keywords:** COVID-19, neoplasms, children, paediatric, policy, global

## Abstract

The COVID-19 pandemic poses an unprecedented health crisis in all socio-economic regions across the globe. While the pandemic has had a profound impact on access to and delivery of health care by all services, it has been particularly disruptive for the care of patients with life-threatening noncommunicable diseases (NCDs) such as the treatment of children and young people with cancer.

The reduction in child mortality from preventable causes over the last 50 years has seen childhood cancer emerge as a major unmet health care need. Whilst survival rates of 85% have been achieved in high income countries, this has not yet been translated into similar outcomes for children with cancer in resource-limited settings where survival averages 30%. Launched in 2018, by the World Health Organization (WHO), the Global Initiative for Childhood Cancer (GICC) is a pivotal effort by the international community to achieve at least 60% survival for children with cancer by 2030.

The WHO GICC is already making an impact in many countries but the disruption of cancer care during the COVID-19 pandemic threatens to set back this global effort to improve the outcome for children with cancer, wherever they may live.

As representatives of the global community committed to fostering the goals of the GICC, we applaud the WHO response to the COVID-19 pandemic, in particular we support the WHO’s call to ensure the needs of patients with life threatening NCDs including cancer are not compromised during the pandemic. Here, as collaborative partners in the GICC, we highlight specific areas of focus that need to be addressed to ensure the immediate care of children and adolescents with cancer is not disrupted during the pandemic; and measures to sustain the development of cancer care so the long-term goals of the GICC are not lost during this global health crisis.

## Introduction

The success of global public health measures over the last 50 years, particularly childhood immunisation, improved nutrition and infection prevention, has led to a marked global reduction in early childhood mortality. However, wherever the health of children improves from preventable causes, cancer in childhood inevitably emerges as a significant and unmet health care need [1].

Collectively, childhood cancers represent a major global child health burden. Each year at least 400,000 children and adolescents in the 0– 19 age group develop cancer, a figure that is probably a serious underestimate due to a worldwide lack of access to cancer diagnosis and treatment, and the lack of systematic cancer registration in many settings [2–4].

Cancers occurring during childhood and adolescence are clinically and biologically distinct from those seen in adults; and combined with their special developmental needs, children and adolescents with cancer have unique demands and experiences across the cancer journey. Over the last 50 years, the development of age-appropriate multidisciplinary care and cancer treatment based on research-informed international collaborative clinical trials have achieved a remarkable improvement in survival for children with cancer. These outcome benefits need not be limited to high-income countries; evidence shows that cancer treatment for children in many low- and middle-income countries is both cost effective and can be achieved with simple, safe and affordable treatment protocols [1, 4, 5].

Launched in 2018, the World Health Organization (WHO) Global Initiative for Childhood Cancer (GICC) has mobilised the global child cancer community in a focused effort to improve the survival of children with cancer to over 60% by 2030; this truly transformative change has an initial focus on six common cancers: Acute lymphoblastic leukaemia, Burkitt lymphoma, Hodgkin lymphoma, Retinoblastoma, Wilms tumour and Low-grade glioma, and may save over 1 million lives by the turn of the next decade [6].

Unfortunately, the serious impact the COVID-19 pandemic (declared on 11 March 2020) has had on public health services worldwide presents a major barrier and a global challenge to maintaining the gains already achieved and may seriously impair future progress towards the long-term goals of the GICC.

Healthcare professionals caring for children and adolescents with cancer applaud the worldwide mobilisation of efforts to mitigate the SARS CoV-2 virus and treat those affected with COVID-19. However, we implore all agencies and health services to collaborate and ensure the current and future needs of children and adolescents with cancer are considered across all national and regional initiatives to respond to the COVID-19 pandemic.

This quest is not just for access to early diagnosis, cancer care and treatment, but also for improved and sustained access to the psychosocial support families require to ensure their child can be treated in a timely manner. Moreover, for the many children and young people worldwide, who have life limiting diagnoses, we implore health services and health ministries to ensure timely access to palliative care especially the medication needed for the relief of pain and suffering.

## Challenges posed by the COVID-19 pandemic

A survey of 155 countries released by the WHO on 1 June 2020 [7] shows the breadth of impact on health services for those with cancer and other noncommunicable diseases (NCDs), including the cancellation of elective health services (especially surgery and radiotherapy), shortages of essential medicines and delayed diagnostics, the overwhelming of hospital inpatient services and cancellation of essential outpatient services. This has been compounded by serious and ongoing healthcare staffing issues due to the direct and personal impact of COVID-19 on the health and wellbeing of nursing, medical and allied professionals and their families. The WHO Pulse Survey released in August 2020 confirmed this concerning trend across multiple disease areas [8].

Unlike a medical emergency where a life may be saved immediately, saving the life of a child or young person with cancer is a marathon, not a sprint, it takes many months of sustained and coordinated effort by multiple healthcare teams, and the child’s family, to achieve a life free of cancer.

Thus, the disruption of health services during this pandemic presents a serious challenge to maintaining and improving the care of children and adolescents with cancer, where equitable access to safe and affordable treatment is the cornerstone of the GICC.

**Figure 1.**
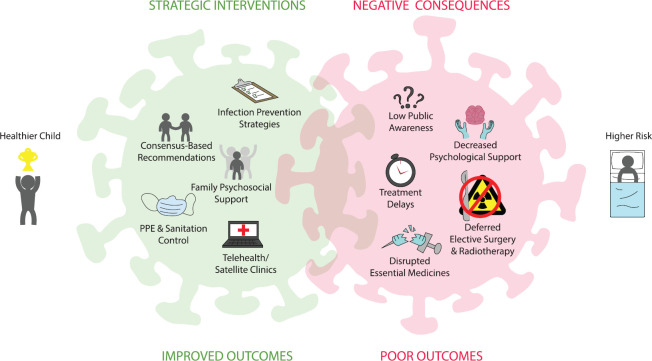
COVID-19 impact on childhood cancer.

Several challenges presented by the COVID-19 pandemic are particularly relevant to the care of children and adolescents with cancer (Figure 1). We list them below with proposed mitigation strategies.

### Reduced public health awareness of cancer

The developmental origins of childhood cancers and their presentation in early life preclude public health measures at primary prevention. Moreover, many childhood cancers develop rapidly with symptoms and clinical signs in young children, such as those seen in acute lymphoblastic leukaemia, Burkitt lymphoma, that resemble, or occur concurrently, with those from other much more common causes, especially viral, bacterial and parasitic infections. Thus, timely referral for the assessment and investigation of children and adolescents with unexplained symptoms or new clinical signs suggestive of cancer is crucial for making a diagnosis and commencing timely treatment.

However, the observed reduction since the start of the pandemic in referrals of children with cancer symptoms, especially those with solid and brain tumours, will inevitably led to a surge in late-stage diagnosis leading to sub-optimal survival and outcome [9, 10].

***Mitigation strategy:*** Health services and Health Ministries should implement or strengthen public health measures to raise awareness of cancer in children and adolescents. National ‘Early Warning Signs’ programmes are proven to be effective at encouraging parents and local healthcare providers to seek advice and early referral for children with possible cancer [11–14].

Parents need reassurance that it is both necessary and safe for them to have their child’s symptoms investigated as soon as possible and practical.

### Treatment interruption, treatment delays and modification

The cure of cancer in children and adolescents is achieved by the timely and coordinated delivery of multimodal treatment including chemotherapy, surgery and radiotherapy (where indicated). The key to the successful treatment of childhood cancer is its sequencing and cadence; this means maintaining the intensity of treatment, with minimal disruption or delay to systemic chemotherapy, and the delivery of intercalated local therapy with surgery and/or radiotherapy. Delayed, interrupted or modified chemotherapy, deferred surgery and failure to provide appropriately timed radiotherapy reduce the chance of achieving disease remission, increase the chance of treatment failure and the raise the prospect of a premature or preventable death.

In response to the pandemic emergency, the International Society of Paediatric Oncology (SIOP) collaborated with St Jude Children’s Research Hospital to establish an International COVID-19 Registry to record prospectively the impact of COVID-19 infection in children with cancer.

As of October 2020, the 30-day mortality of the 730 cases of children with cancer with complete follow-up was 5% [15] and significantly higher than children without cancer. Of equal concern is the observation from surveys of paediatric oncology services that planned treatments for non-COVID-19 infected patients on active treatment are withheld, unavailable or postponed despite recommendations of the expert consensus group and parent organisations that safe and effective treatment should be continued unmodified wherever possible [9, 16].

***Mitigation strategy:*** It is crucially important that health care providers maintain and support cancer services and ensure they can provide consistent and timely multimodal treatment while protecting patients and staff from infection [18–21]. Collaborative data registries, such as the International COVID-19 Registry, can provide the evidence needed for assessing the impact of the pandemic on cancer services and identify areas needing intervention. Consensus expert guidance and recommendations have been published previously from international organisations [16]: any local or regional adaptation of treatment due to shortages of essential medicines or other resource constraints should be limited and wherever possible and agreed by the local healthcare teams in consultation with parents.

### Decreased psychosocial support for parents, families and healthcare staff

The true impact of the pandemic on the families of children with cancer is unknown but anecdotal experience across the globe suggests the effect has been severe and has brought the need for access to psychosocial support to the fore. Moreover, the access and availability of palliative care support and symptoms relief for end-of-life care also appears to have been severely compromised during the pandemic.

Without affordable access to public transport or money to pay for travel, many families face the stark reality of their child not receiving any treatment for cancer. For families with little or no income, the need to travel long distances for treatment without any support for accommodation, or access to food, and other essentials is the source of extreme stress, raising the risk of treatment abandonment [22], medically induced poverty and preventable death.

***Mitigation strategy:*** Healthcare teams, health services and support organisations need to ensure families have access to ongoing psychosocial support, during and after the pandemic.

Where children and adolescents develop life-limiting cancer or end-of-life complications, access to palliative care including access to essential medications for pain and symptom relief is vital for compassionate treatment and comfort care.

It is also essential for nursing, medical and allied health staff to be provided with ongoing support by their employers for they face the dual challenge of caring for their patients while needing to care for themselves and their own families during this crisis.

### Disruption to essential cancer medicines access and supply

Of urgent concern is the anticipated disruption to supply and ongoing shortages of essential cancer chemotherapy and supportive care medicines which are the mainstay of curative treatment in children [7, 23]. The supply and distribution of essential cancer medicines has always been fragile and erratic in many resource-limited settings and this has been compounded by the need to redirect limited funding and ‘manpower’ to the supply of personal protective equipment (PPE) and other consumables for the care of adult patients with COVID-19.

Efforts to ensure access to drugs on the WHO Essential Medicines List for children and those proposed in the paediatric review of the Essential Medicines List for cancer [24, 25] are vital to ensure curative care can be delivered to as many children in as many clinical settings as possible.

***Mitigation strategy:*** Government Health Ministries need to ensure their national approved drug lists include those required to treat children (and adults) with cancer, and their drug regulatory authorities and purchasing agencies follow rigorous but streamlined regulatory process and pharmacovigilance to achieve sustained access to quality and affordable cancer chemotherapy. Special attention is required for the purchase and delivery of Asparaginase, a temperature sensitive therapeutic protein that is crucial for the cure of acute lymphoblastic leukaemia, which accounts for one third of all cases of cancer in childhood. Disrupted or delayed delivery, and loss of the cold-chain, could cause an unmeasured loss of activity and effectiveness for the vital cancer agent.

### Delayed diagnostic procedures and ‘local therapy’ with surgery and radiotherapy

Few childhood cancers, aside from the acute leukaemias and Non-Hodgkin Lymphoma, can be cured with chemotherapy alone, and almost all childhood solid tumours and brain tumours require ‘local therapy’—either surgery and/or radiotherapy—to either resect or irradiate the primary tumour. The diagnosis and staging of most childhood cancers involves diagnostic imaging and interventional biopsies often under general anaesthetic. The surgical resection of primary solid tumours in children may occur at diagnosis, but most often surgery follows a course of preoperative chemotherapy and may then be followed by radiotherapy and further chemotherapy. Access to diagnostic imaging and procedures for disease staging, and timely well-planned surgery and radiotherapy are crucial for achieving curative outcomes for many children and adolescents with cancer.

The evidence of the impact of COVID-19 pandemic on the initial diagnosis and staging of children with cancer and access to ‘local therapy’ is emerging [26–29]. However, many centres are overwhelmed by the demand of COVID-19 patients, coupled with the need to minimise the risk of healthcare teams and patients to infection, and have severely restricted access to, or delayed and deferred, scheduled surgical procedures and radiotherapy. The true long-term impact of these reduced services will be difficult to gauge especially the impact on the survival of children where there have been delays in local therapy for solid tumours.

***Mitigation strategy:*** As regions begin to emerge from the COVID-19 crisis, the early return of full scheduled surgery lists and radiotherapy treatment will be essential for the continuity of care for children and adolescents with cancer. Planned cancer therapies, including radiation therapy should never be considered ‘elective’, since delays and gaps in care increase rate of relapse and decrease overall survival.

## Towards collaborative solutions for emerging from the pandemic

The global community of child cancer healthcare professionals welcomed the statement of Dr Tedros Adhanom Ghebreyesus, Director-General of the WHO, affirming that ‘It’s vital that countries find innovative ways to ensure that essential services for NCDs continue, even as they fight COVID-19’ [30]. The role of the WHO and member states in addressing the major health needs during this pandemic cannot be overstated. As international organisations dedicated to the care of children with cancer, we offer our support, advocacy and collaboration as we seek solutions on behalf of our patients, their families and our healthcare teams to this global crisis.

We strongly advocate for the engagement of health services and health ministries with childhood cancer professionals and parent support organisations in the development and implementation of regional COVID-19 response strategies as we continue and emerge from the current pandemic and other health emergencies potentially arising in the future. It is also essential that pandemic crisis management has a connection with health care planning for NCDs, including paediatric cancer control. As stakeholders in child and adolescent cancer care, we support the implementation of COVID-19-specific strategies and their integration with NCD action plans at all governance levels. Such strategies have relevance during and beyond the pandemic to ensure the momentum of the WHO GICC is maintained.

### Regional response strategies where we can offer specific support

**SARS-Cov2-specific measures:**

- The accrual of objective evidence from multi-stakeholders and the broad community of the impact of COVID-19 to guide the mitigation of COVID-19 on childhood cancer treatment.
- The development of a reporting framework and research agenda on the impact of COVID-19 on childhood cancer care delivery, patient outcomes and workforce at all resource levels, with the aim to develop evidence-based best practice for clinical and care organisation decision-making.
- The development of consensus-based recommendations during the pandemic for the ongoing care across all aspects of the paediatric cancer patient pathway, including the well-being of health care workers by promoting infection prevention strategies and mental health support.
- The development of evidence on strategies and guidelines for physicians, nurses and support staff to mitigate the risk of COVID-19 transmission within the community, during transportation and at the medical facilities. These include isolation and screening procedures for patients, families and visitors, quarantine for COVID-19-positive patients (while maintaining access to oncology services, support during the infection and continuation of cancer-curing therapies), staff training on PPE and infection control, cleaning and sanitation guidelines, deferment of non-urgent hospital and clinic visits using telehealth, satellite clinics, family psychosocial support and increased financial and logistical assistance for families in crisis.

**Evidence-based adaptations to cancer care in high COVID-19 prevalence communities**

- Provide guidance on the adaptation of treatment regimens across settings during the pandemic.
- Highlight unmet needs in areas adjacent to care delivery, such as support of transportation and housing for families staying with their severely ill children during treatment, particularly in low- and middle-income countries.
- Consider the needs of children and young people with cancer in national cancer planning for COVID-19 recovery.

**Ongoing evidence-based activities that must not be suspended during the pandemic and for which the pandemic may provide additional impetus for action**

- Implementation of national awareness of childhood cancer early warning signs to ensure timely referral of children with suspected cancer, which could be combined with education about COVID-19 prevention and control.
- Diversification and reinforcement of global medication supply chains and streamlined regulatory procedures.
- Contingency planning for all critical oncology infrastructure in cases of outages. Radiation therapy, diagnostic imaging and other procedures that depend on potentially fragile equipment may lack access to routine service and maintenance if the service contractor is immobilised by the pandemic. Contingency planning and pre-established relationships with other facilities to provide services during outages not only protect patients during a pandemic, but during outages that occur for any other reason.
- Support institutional and government decisions on maintaining paediatric cancer as a priority within hospital structure, and preventing unnecessary delays in treatment (chemotherapy, surgery, radiotherapy, supportive and palliative care). If for any reason, delays should be necessary, they should be planned in a way as not to have impact on survival.

As we emerge from the pandemic health crisis, we strongly support the implementation of the WHO GICC. We will advocate with Health Ministries and Health Services to adopt recommendations in the WHO technical packages for the development of child and adolescent cancer services.

In particular we support the:

- Development of national child and adolescent cancer control plans.
- Sustained procurement and supply of essential cancer chemotherapy and supportive care medicines, including blood products, and medical devices
- Maintenance of the momentum of the WHO GICC across all global regions in partnership with the WHO and other agencies.

## Conclusion

As representatives of a wide range of childhood cancer health professionals and researchers, expert parent and survivor NGOs, and advocates, the global paediatric cancer community stands ready to partner with national health services and government ministers in COVID-19 mitigation programmes that promote the well-being of all children and adolescents, including those with cancer as a particularly vulnerable population with specific needs. We also wish to look beyond the current pandemic and support the wide implementation of the WHO GICC, to cure more and care for all, wherever they may live. We welcome the ongoing efforts of the WHO NCD Directorate to accelerate implementation of the GICC during 2021, taking into account COVID-19 mitigation strategies, and we will continue to be partners in supporting implementing countries.

## Conflicts of interest

No authors declare any conflicts of interest in relation to the contents of this article.

## Funding statement

No specific funding was received for this work.
